# Cytotoxic and Proapoptotic Effects of Resveratrol in In Vitro Studies on Selected Types of Gastrointestinal Cancers

**DOI:** 10.3390/molecules26144350

**Published:** 2021-07-18

**Authors:** Katarzyna Ratajczak, Sylwia Borska

**Affiliations:** Department of Histology and Embryology, Department of Human Morphology and Embryology, Faculty of Medicine, Wroclaw Medical University, 50-368 Wroclaw, Poland; sylwia.borska@umed.wroc.pl

**Keywords:** resveratrol, apoptosis, proliferation, multidrug resistance (MDR), anticancer therapy, gastrointestinal cancers

## Abstract

Cancer diseases are currently one of the greatest health challenges in clinical medicine worldwide. Classic methods of treatment often lead to numerous side effects, including the development of multidrug resistance. For this reason, increasing hope is being placed on compounds of natural origin, mainly due to their pleiotropic effect on different types of cells, protective effect on normal cells and toxic effect on cancerous ones. The most studied group are the polyphenolic compounds, which include resveratrol. The effectiveness of polyphenols in the treatment and prevention of many diseases, including cancer of various origins, has become the basis of many scientific studies. The anticancer effect of resveratrol has been demonstrated at all stages of the carcinogenesis process. Additionally, whether administered by itself or in combination with cytostatics, it may play a significant role in the process of reversing multidrug resistance. A review of the effects of resveratrol in in vitro conditions proves that it has a stronger or weaker antiproliferative and proapoptotic effect on the cells of certain neoplasms of the gastrointestinal tract. Despite the differences in the effect of this compound on different types of cancer, a similar tendency can be observed especially regarding the correlation between the concentration of the compound and the incubation time on the one hand and the antitumour effect on the other hand. The information included in this review may prove helpful in planning in vivo and clinical studies in the future.

## 1. Introduction

The progress in medicine in terms of prevention programs, diagnostic tests and therapeutic strategies, as well as the awareness of industrialized societies in relation to the importance of physical activity and proper nutrition, have contributed to a significant improvement in the quality of life and the average life expectancy. Unfortunately, this is connected with an increasing incidence of aging-associated diseases, including cancer.

Despite the implementation of cancer prevention, the incidence and mortality rates are constantly increasing everywhere in the world [1,2]. As a rule, only early detection of neoplastic changes allows for the introduction of effective treatment, significantly increasing the chances of complete remission and the patient’s survival.

Gastrointestinal cancers, due to the lack of clear and characteristic symptoms, are most often diagnosed at a late stage of the development of the disease. The presence of metastases makes the treatment a long-term process which hardly ever brings the desired therapeutic effect. The factors influencing the formation and development of neoplastic diseases include, among others, lifestyle and diet. On the basis of numerous studies, it has been shown that the use of an appropriate and balanced diet rich in vegetables and fruit can contribute to reducing the risk of cancer [3,4,5]. For this reason, increasing attention is being paid to compounds of natural origin that have been present in medicine for hundreds of years. Paradoxically, the development of modern medicine has led to cancer’s gradual elimination by chemically synthesized compounds. In the case of chemotherapy, the drugs used show toxicity towards organs and often cause the emergence of resistance, which causes the lack of an adequate response to the treatment [6]. On the other hand, the substances used to reduce multidrug resistance (MDR) have additional side effects. MDR in the context of cancers diseases is an unfavourable phenomenon that contributes to the failure of the use of anticancer therapies. It is defined as the insensitivity of the tumour cells to groups of various therapeutic agents and develops as a result of using a single cytostatic drug [7]. The classic mechanism of MDR is related to the overexpression of the *ABCB1* gene, encoding the P-glycoprotein (P-gp) protein, through which the drug is transported outside the cell, leading to a reduction in the effective concentration of the drug in the cell [8,9]. In addition to the classic mechanism of multidrug resistance, there are also atypical mechanisms related to gene overexpression: *ANXA1* and *TXN* or topoisomerase II—an enzyme that plays an important role in replication, transcription and recombination, as well as in the construction and segregation of chromosomes. The *ANXA1* gene codes for the protein annexin I, which is involved in the regulation of cell growth, differentiation and apoptosis [10]. The *TXN* gene encodes the thioredoxin protein, which is involved in the regulation of the redox state of the cell. As an example of a breaking phenomenon of MDR by resveratrol, studies were conducted on stomach cancer cell lines sensitive and resistant to the action of suitable cytostatics: EPG85-257P line (sensitive to both cytostatics), EPG85-257RDB line (resistant to daunorubicin) and EPG85-257RNOV line (resistant to mitoxantrone). The action of resveratrol led to the sensitization of cancer cells to the action of chemotherapeutic agents by influencing the expression of genes (*ABCB1*, *ANXA1*, *TXN)* and proteins (P-gp, annexin I, thioredoxin) related to the MDR process [11]. The multidirectional effect of resveratrol on the mechanisms of MDR was also demonstrated in pancreatic cancer cell lines: EPP85-181P (sensitive to cytostatics), EPP85-181RDB (resistant to daunorubicin) and EPP85-181RNOV (resistant to mitoxantrone). In EPP85-181RDB cells, resveratrol reduced the level of P-gp expression, while in EPP85-181RNOV cells, resveratrol increased the expression of topoisomerase II [12]. Hence, increasing hope is being placed on compounds of natural origin such as plant polyphenols, which, when administered in a monitored manner, are not toxic to healthy organs. This is evidenced by numerous scientific studies that document the anticarcinogenic properties of polyphenols, which are important in the prevention and treatment of cancer diseases [13,14,15,16,17]. Natural substances are a rich source of compounds with various biological properties, such as antibacterial, anti-inflammatory and anticarcinogenic properties, thus offering more comprehensive therapeutic effects than individual drugs [18,19]. Naturally occurring polyphenolic compounds are secondary metabolites produced by plants in order to protect them against stress factors from the natural environment, such as UV radiation and viral and fungal infections [20,21].

**Figure 1:**
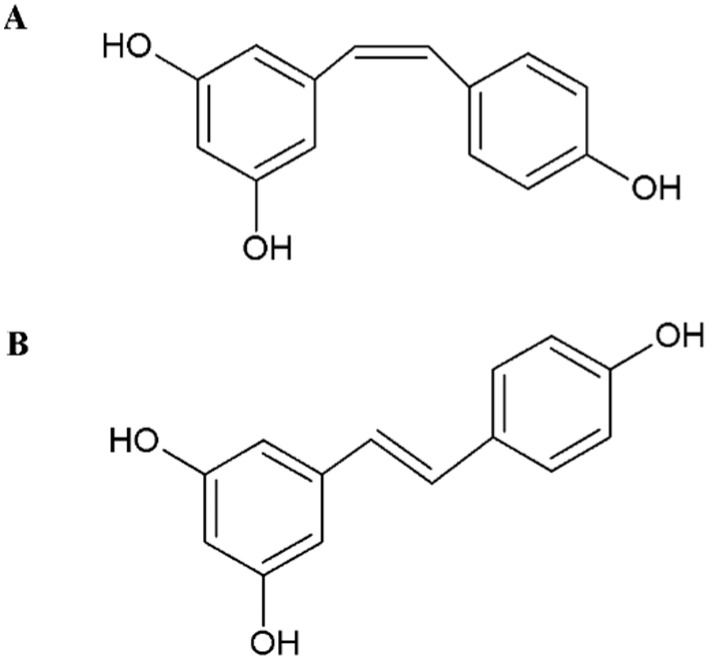
Chemical structure of *cis*-(**A**) and *trans*-resveratrol (**B**).

**Figure 2:**
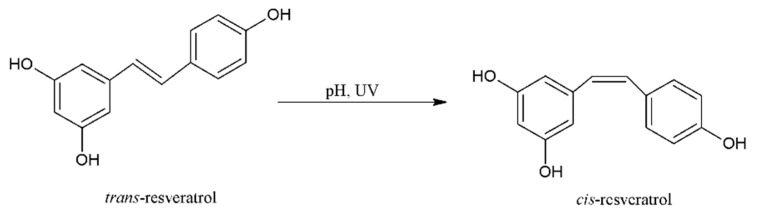
Isomerisation of resveratrol.

One of the plant polyphenols more often studied is resveratrol. Resveratrol (3,5,4’-trihydroxy-trans-stilbene) is a representative of stilbenes, which are small-molecule compounds containing two aromatic rings in their structure. Resveratrol was first isolated in 1940 from the roots of white hellebore (*Veratrum grandiflorum*) [15]. Depending on the attachment position of individual functional groups and their mutual position in relation to each other and in relation to the double bond, these compounds exist in two stereoisomeric forms: *cis*-resveratrol and *trans*-resveratrol (Figure 1) [22]. Resveratrol is found in both isomeric forms. However, the steric point of view, the *trans* isomer is more stable than the *cis* isomer. In addition, the *trans*-resveratrol form is better researched and shows greater activity compared with the *cis* form. Moreover, *trans*-resveratrol is more biologically active, for example, in its antioxidant and anticancer properties [23,24,25]. The *cis* and *trans* isomers coexist in plants and wine. However, the *cis*-resveratrol isomer has never been found in grape extract [26]. The *cis* form is formed by isomerization through the action of UV radiation, artificial light, high pH on the *trans* form or during the fermentation of grape skins (Figure 2) [27,28].

Since then, this compound has been extracted from many other naturally occurring plants included in a varied diet, including bilberries, blueberries and peanuts [29,30]. However, its main source is the skin of dark grapes (50–100 µg/g) [31,32].

**Figure 3:**
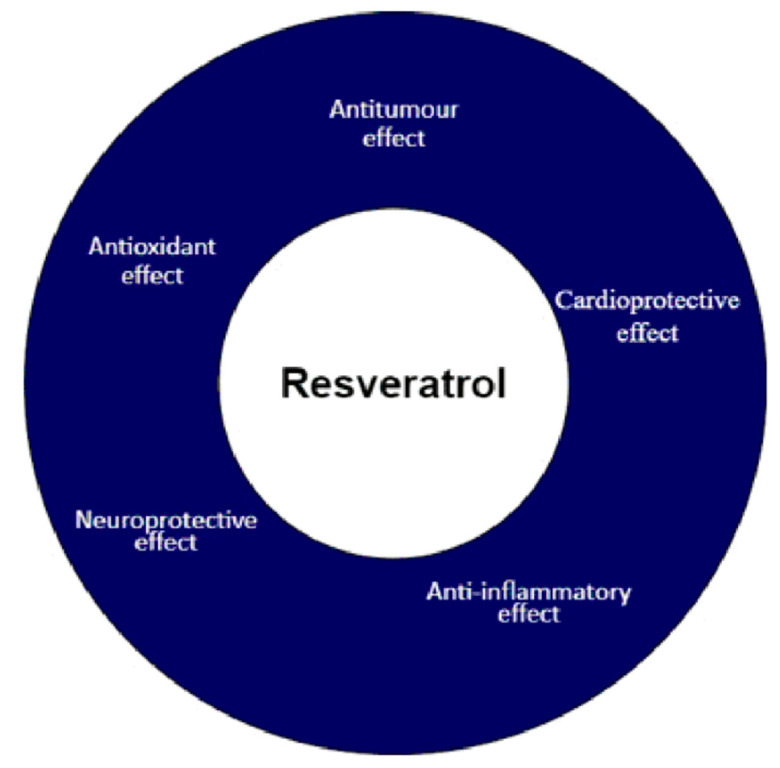
Potential clinical properties of resveratrol.

Resveratrol is a phytoalexin, a compound produced by plants in response to the attack of pathogens and as a result of UV radiation and the action of heavy metals [33,34]. For this reason this compound exhibits several diverse biological activities (Figure 3) [35]. In France, despite limited physical activity, high consumption of animal fats and substantial consumption of red wine, a low mortality rate from cardiovascular disease is observed. This phenomenon is known as the French paradox. The association of the presence of resveratrol in red wine with its cardioprotective effect resulted in a great interest in this compound [36]. The anti-inflammatory effect of resveratrol is due to its ability to inhibit the synthesis and secretion of inflammatory mediators [37]. Resveratrol also has a neuroprotective effect on the brain. These properties are used in the treatment of many neurodegenerative diseases, such as Alzheimer’s disease or Parkinson’s disease [38]. The antioxidant effect of resveratrol is related to the inhibition of the formation of free radicals and the removal of those already formed from the body [39]. In our review, we focus on the antitumour activity of resveratrol.

The antitumour activity of resveratrol was first described in 1997 by Jang et al. [40,41,42]. Due to the risk associated with traditional methods of cancer treatment, the use of this compound in cancer patients is particularly important, something that has been confirmed by numerous studies on the relationship between resveratrol and the protection against the side effects of chemotherapy [32]. The in vitro studies conducted so far have proven that resveratrol exhibits antitumour activity at all stages of the carcinogenesis process [43]. However, the mechanism of action of this compound is still not fully understood. Moreover, the effect of resveratrol varies depending on factors such as the type of cancer, the concentration and the duration of the action. The healing properties of resveratrol can be observed in many types of cancer, including breast, skin, intestine, prostate and lung cancers [44,45,46,47]. Additionally, resveratrol participates in the process of overcoming multidrug resistance by changing the expression of the genes and proteins related to the MDR phenomenon and by sensitizing neoplastic cells to the action of chemotherapy agents [11], [48].

The main goal of this review is to present the results of in vitro studies on the antiproliferative and proapoptotic effects of resveratrol on human gastrointestinal cancers.

## 2. Pancreatic Cancer

Pancreatic cancer is the ninth most common cancer in women and the tenth most common cancer in men [49,50]. The highest number of cases is recorded among the elderly [51,52]. Pancreatic cancer is difficult to diagnose due to the lack of characteristic symptoms [53]. Moreover, the lack of appropriate and cost-effective screening tests allowing for early detection of this cancer leads to its detection in the advanced stage of the disease. Currently, the most feasible treatment option for pancreatic cancer is surgery performed at an early stage of the disease [54]. Late diagnosis significantly affects poor prognosis in patients due to metastases and the development of MDR, which is the cause of chemotherapy failure [55,56]. Additionally, the selection of an appropriate treatment depends on the patient’s physical condition, including the presence of other diseases [51].

### 2.1. Antiproliferative Effect

In the case of pancreatic cancers, the results of numerous in vitro studies indicate the potential antiproliferative effect of resveratrol, depending on the dose and the time of incubation with the compound.

In a study on two pancreatic cancer cell lines, PANC-1 and AsPC-1, Xian-Zhong Ding’s team showed that after 48 and 72 h of incubation with a resveratrol solution at various concentrations, there was a statistically significant change in cell viability. These changes were dependent on the time and concentration of the compound tested. The greatest effects were observed at a concentration of 100 µM [57]. However, these studies did not show the effect of this compound on normal pancreatic cells.

The studies of Jing Cui’s team also showed an inhibition of cancer cell survival dependent on the concentration and time of the effect of resveratrol. The tests were performed on the PANC-1, BxPC-3 and AsPC-1 pancreatic cancer lines, which were treated with resveratrol in a concentration range from 0 to 200 µM/L at 24, 48 and 72 h [58]. Studies on the effects of resveratrol in the same cell lines made by scientists at the Tongji University also showed its inhibitory effect on cell proliferation. The cells were incubated with resveratrol at concentrations ranging from 25 to 250 µM/L at 24, 48 and 72 h [59].

Another neoplastic model of pancreatic cancer—the Capan-2 line—was also used in order to study the effect of resveratrol on cell survival. After 24 h of exposure of the cells to resveratrol (10, 50, 100 µM), a concentration-dependent decrease in cell viability was observed [60]. The studies did not take into account the effect of the compound on normal pancreatic cells.

The effect of resveratrol on cell survival was also studied by P. Liu’s team. Three pancreatic tumour lines were used in the study: PANC-1, CFPAC-1 and MIA PaCa-2, as well as normal pancreatic cells derived from tissue collected from a patient during surgery. The cells of the lines tested were exposed for 72 h to various concentrations of resveratrol. An increased cell growth inhibition was observed to take place with an increasing compound concentration. Compared with neoplastic cells, normal pancreatic cells showed greater resistance to the cytotoxic effect of resveratrol [61]. The cell lines differed from one another in their sensitivity to the effects of polyphenol. Lines PANC-1 and MIA PaCa-2 were the most sensitive to the effects of resveratrol. The AsPC-1 line showed moderate sensitivity to the action of the compound. However, the lowest effects of the action of the compound were recorded for the Hs766T line [62].

Most studies confirm that the higher the dose of resveratrol, the stronger its antiproliferative effect on cells of various pancreatic cancer lines. At the same time, such a strong effect is not observed in normal pancreatic cells, which may translate into resveratrol being an effective and safe treatment option in the future.

### 2.2. Proapoptotic Effect

Apoptosis is a complex physiological process that follows a specific pattern that significantly influences the proper functioning of the organism. This process leads to the removal of unnecessary and damaged cells that could potentially pose a threat to the organism (e.g., cancer cells) [63]. Due to its essential importance in the tumorigenesis process, apoptosis is a topic that has been frequently explored in research.

For pancreatic cancer, resveratrol has been shown to be proapoptotic in many cases. In the studies of Xian-Zhong Ding’s team, the ability of resveratrol to induce apoptosis was examined by using flow cytometry. The pancreatic tumour cell lines PANC-1 and AsPC-1 were subjected to 72 h of incubation with 100 µM/L resveratrol. After this time, the induction of the apoptotic process was 27% and 34% for PANC-1 and AsPC-1, respectively [57].

Similar tests were performed by Jing Cui et al. The cells of three pancreatic cancer lines (PANC-1, AsPC-1 and BxPC-3) were incubated in resveratrol for 48 h at concentrations ranging from 0 to 200 µM/L. The analysis of the results showed that the induction of the apoptotic process was 26.20% for the PANC-1 cell line. A similar value was found for the BxPC-3 line: 22.08%. As for the AsPC-1 line, the percentage increase in apoptotic cells was only observed at a concentration above 150 µM/L [58]. The team at Tongji University obtained similar results for the same cell models. During a 24 h incubation with resveratrol at concentrations ranging from 0 to 200 µM, it was shown that the percentage of apoptotic cells at a concentration of 100 µM was 16.2%, 18.21% and 22.26% for the PANC-1, AsPC-1 and BxPC-3 lines, respectively [58].

Studies on resveratrol’s ability to induce the apoptotic process performed on the Capan-2 cancer cell line showed that the number of apoptotic cells after 24 h of incubation with 100 µM/L resveratrol solution increased significantly (from 12.92% to 21.31%) compared with cells not treated with the compound [60].

The effect of resveratrol on apoptosis was also demonstrated by P. Liu’s team. Research shows that microRNAs can act as oncogenes or tumour suppressors; hence, they play an important role in the process of cancer initiation and progression [64]. One of the most frequently expressed miRNAs in tumours is miR-21. According to data in the literature, miR-21 influences the level of Bcl-2, a key apoptotic regulator [65]. Three pancreatic tumour lines were used in the studies: PANC-1, CEPAC-1 and MIA PaCa-2. Cells were treated with resveratrol at a concentration of 50 µM for 24 h. A real-time PCR was then performed to check for changes in miR-21 expression following the treatment of the cells with the compound. It turned out that there was a statistically significant reduction in the level of miR-21 against the control. The inhibition of the miR-21 expression resulted in a reduction of the Bcl-2 protein expression [61].

The effect of resveratrol on the activation of the executioner caspase-3, which is normally involved in typical apoptosis, was also studied. The tests were carried out on four pancreatic cell lines: PANC-1, MIA PaCa-2, Hs 766T and AsPC-1. The cells were incubated for 48 h with resveratrol (0–40 µM). Caspase-3 activity, which was measured with the help of a fluorometer, was the lowest for the Hs 766T line, while the highest activity was observed for the PANC-1 and the MIA PaCa-2 lines. For the AsPC-1 line, caspase-3 activity was intermediate in relation to the remaining cell lines. These studies did not include a reference to a normal pancreatic cell line [62].

In conclusion, resveratrol is a proapoptotic compound in human pancreatic cancer, and it acts through many different pathways involved in this process, especially at high doses.

## 3. Stomach Cancer

Despite the observed reduction in the incidence of stomach cancer, it remains one of the most common neoplasms, especially among men [66]. Similar to pancreatic cancer, stomach cancer is typically diagnosed at a late stage due to the lack of early characteristic symptoms [67]. A late diagnosis often limits the use of effective treatments, something that is associated with a poor prognosis. The causes of stomach cancer are not clearly defined. However, it is known that in the case of gastrointestinal cancers, the type of diet is an important factor [67]. Epidemiological studies have shown that the risk of developing the disease is significantly reduced when the diet includes fruits and vegetables containing large amounts of polyphenols [66,67,68].

### 3.1. Antiproliferative Effect

Separate reports from recent years indicate that resveratrol is a compound that is also used in the treatment of gastric cancer [69].

Xiaoping et al. analysed in vitro the effect of resveratrol on the survival of human gastric cancer cells as well as its potential mechanism of action. For this purpose, MGC-803 cells were exposed to different concentrations of resveratrol for 24 and 48 h. In this experiment, resveratrol was shown to inhibit cell growth depending on the dose and the duration of the effect. Accordingly, further experiments were performed to evaluate the effect of the compound on the cell cycle. The cells were incubated with resveratrol (50 and 100 µM, 24 h). The analysis showed that the treatment with resveratrol led to the inhibition of the cycle in the G0/G1 phase [70]. Other studies also showed an inhibitory effect of resveratrol on the proliferation of MGC-803 gastric cancer cells. In this case, the treatment performed with concentrations of 50 µM, 75 µM and 100 µM for 24, 48 and 72 h resulted in a time- and dose-dependent decreased cell viability. These changes were statistically significant [71].

Studies on the effect of resveratrol on the inhibition of cell growth were also carried out on the gastric cancer model SGC-7901 cell line. A 48 h cell incubation with resveratrol (0–200 µM) resulted in an inhibition of cell proliferation proportional to an increasing concentration of the compound [72]. Similar studies also performed on the SGC-7901 cell line showed that a 24 h incubation with a resveratrol solution (25 µM and 50 µM) also resulted in a concentration-dependent and statistically significant reduction in cell viability compared with untreated cells [73]. Concentration- and time-dependent inhibition of cell viability in the same cell line was demonstrated by treating it with a resveratrol solution (0–400 µM) for 24, 36 and 48 h [74].

Further studies on the effect of resveratrol on neoplastic cells in gastric cancer were carried out on two cancer cell lines: the aforementioned SGC-7901 line and the BGC-823 line, as well as the benign epithelial cell line GES-1. The cell lines were exposed to various resveratrol concentrations ranging from 0 to 400 µM for 24, 48 and 72 h. A significant reduction in viability was observed in all cell lines tested. These changes were dose- and time-dependent [75].

The proliferative activity of gastric cancer cells is significantly inhibited by resveratrol. The effect of the compound’s action is stronger when the concentration is higher and the exposure time is longer.

### 3.2. Proapoptotic Effect

Studies on the effect of resveratrol on the induction of the apoptotic process were carried out on a gastric cancer cell model: the SGC-7901 cell line. In this experiment, cells were incubated for 48 h with the compound in a concentration ranging from 0 to 200 µM. It was shown that the effect of resveratrol results in an increase in the production of ROS (reactive oxygen species), which according to the literature are involved in the apoptotic process [72]. Additionally, the action of superoxide dismutase and catalase reduced the proapoptotic effects of resveratrol [76]. Yang et al. showed in their studies (also performed on the SGC-7901 cell line) that after 24 h of incubation with a resveratrol solution, the number of apoptotic cells increased significantly in a dose-dependent manner [73]. The same relationship was demonstrated in studies in which cells from the SGC-7901 line were exposed to resveratrol for 24 h at 0, 50, 200 and 400 µM concentrations [74].

As in pancreatic cancer, resveratrol also has a proapoptotic effect in gastric cancer, and the effect observed is stronger when the concentration of the compound is higher. However, there is less documented research on this tumour model.

## 4. Liver Cancer

Liver cancer is also one of the most common cancers worldwide. It is diagnosed more often in men than in women [77]. Despite the improvement of conventional treatment methods, including surgery, chemotherapy and radiotherapy, liver cancer remains a difficult cancer to treat [77]. Similar to other tumours of the gastrointestinal tract, it is typically first diagnosed at an advanced stage, which usually translates into high mortality among patients [77,78]. Resveratrol has an inhibitory effect on liver cancer, which has been confirmed by the results of, among others, in vitro studies.

### 4.1. Antiproliferative Effect

The effect of resveratrol on cell viability was also studied in a liver cell model: the HepG2 line. In the experiment, cells were exposed to resveratrol (0–100 µM) for 24 and 48 h. The results of an MTT test showed a concentration- and time-dependent reduction of the viability in the cell line tested [79]. P.-L. Kuo’s team obtained similar test results. In this case, the study model consisted of two cell lines: HepG2–p53(+) and Hep3B–p53(−). In the experiments, the cells were treated with resveratrol in concentrations of 1, 5, 10 and 20 µg/mL. The cell incubation times with the compound were 12, 24, 48 and 72 h. In the case of the HepG2 line, treatment with resveratrol resulted in a statistically significant reduction in cell viability. These changes were dependent on the concentration and the duration of the action of the compound. For the Hep3B line, no statistically significant changes in cell viability were found [80]. Additionally, an analysis of the changes in the cell cycle after incubation with resveratrol was performed for the HepG2 line. It was shown that at concentrations of 10 and 20 µg/mL during a 24 h incubation, cells accumulate in the G1 phase. The most visible changes were observed at a concentration of 20 µg/mL. The increase in G1 cell population resulted in a decrease in G2 cell population. However, in the S phase, no significant changes in the population of HepG2 cells were observed [81]. In subsequent studies on the effect of resveratrol on cell proliferation, in addition to the HepG2 line, the Bel-7402 and SMMC-7721 cell lines as well as the normal liver cell line HL-7702 were used. The studies showed that a 24 h incubation with the compound (0–200 µM) resulted in the inhibition of the tumour cells compared with normal cells. This effect was seen at resveratrol concentrations higher than 80 µM. These results indicate that resveratrol reduces cell viability depending on the dose [81]. In an experiment performed on the human liver cancer cell line MHCC97-H, a time- and concentration-dependent effect of resveratrol on the inhibition of the proliferation of the cells tested was observed. This effect could be seen during a 24 h and 48 h incubation of the cells with the compound (0, 20, 60 and 100 µM) [82].

In conclusion, the tests performed on liver cancer cell models indicate the antiproliferative properties of resveratrol, primarily the inhibition of the cell cycle. The results are dependent on the concentration and time of the incubation with the compound, as well as the tissue origin of the cells tested.

### 4.2. Proapoptotic Effect

The mechanism of apoptotic induction by resveratrol in liver cancer cells in vitro has not been fully understood yet. However, it is known that the effect depends on the type of cancer lines studied, among which there are differences in the response to the effect of the compound.

In the case of HepG2 cells, P.-L. Kuo’s team showed that resveratrol inhibits cell growth and induces the apoptotic process. The level of p53 protein in the HepG2 and Hep3B cell lines was analysed after incubation with resveratrol. In the HepG2 cell line, the expression level of this protein increased along with the increase in resveratrol concentration, while in the Hep3B cell line, no significant changes in p53 expression level were observed. These results provide evidence that, in this case, the proapoptotic effect of resveratrol is mediated by the p53 protein [80]. Proteins from the Bcl-2 family participate in the regulation of the apoptotic process [83,84,85]. Bax is a proapoptotic protein, while Bcl-2 is one of the antiapoptotic proteins [84]. The confirmation of the proapoptotic effect of resveratrol in the HepG2 cell line is the increase in the expression of the Bax protein along with the increase in resveratrol concentration. Such a relationship was not observed for the Bcl-2 protein [80].

In other studies performed on the HepG2 cell line, the most pronounced effect on the induction of apoptosis was observed (by flow cytometry) at a concentration of 50 µM during a 48 h incubation with the compound [85].

The TUNEL method was used to evaluate the effect of resveratrol on the induction of apoptosis in the liver cancer cell lines HepG2, Bel-7402 and SMMC-7721. After 24 h of incubation with 100 µM resveratrol solution, it was observed that resveratrol increased apoptosis in relation to untreated cells. Moreover, it was shown that resveratrol also influences the apoptotic proteins Bcl-2 (antiapoptotic) and Bax (proapoptotic), which leads to a decrease in the Bcl-2/Bax ratio [81].

The results of all the studies described above suggest that resveratrol may play a significant role in the apoptosis of liver cancer cells by participating in the regulation of the expression of cycle-suppressor and proapoptotic proteins.

## 5. Intestinal Cancer

Intestinal cancer is a common cancer characterised by a high mortality rate all over the world [86]. It most often affects people between 65 and 74 years of age [87,88]. However, in recent years, an increase in the incidence of the disease has been observed among younger people. In the pathogenesis of this cancer, in addition to genetic and epigenetic factors, environmental factors and poor eating habits also play an important role [89,90]. Many studies indicate that family history of intestinal cancer increases the risk of developing the disease. Therefore, it is recommended that such people be screened first [91]. The use of a high-fat diet and eating too much red meat combined with little physical activity contribute to an increased risk of this type of cancer [91]. Early detection of neoplastic changes during screening tests allows for the implementation of the appropriate treatment, which in most cases brings the desired therapeutic effects.

### 5.1. Antiproliferative Effect

Studies carried out on the intestinal cancer lines HT-29 and WiDr have shown that the effect of resveratrol leads to an increasingly stronger inhibition of cell proliferation as the concentration of the compound increases [92].

In another experiment, the intestinal adenocarcinoma cell lines HCA-17, SW480 and HT-29 were used. The cell lines were exposed to various concentrations of resveratrol. A concentration- and time-dependent inhibition of the number of cells was demonstrated in all the lines tested [93].

Another team’s studies on the HT-29 cell line showed varying resveratrol effects depending on the concentration after 48 h of incubation with the compound. The MTT test showed that lower concentrations of resveratrol (1 and 10 µM) stimulated cell growth, while higher concentrations (25 and 50 µM) inhibited cell proliferation [94]. By extending the duration of the incubation to 96 h, a significant cell growth inhibition was observed, especially at a concentration of 50 µM. The two-phase effect of the compound was not observed in HCT-116 colon cancer cells. In this cell line, treatment with resveratrol resulted in a concentration-dependent reduction in cell viability [95].

The anticancer properties of resveratrol have also been shown in in vitro studies in which the research model was the HCT-116 and Caco-2 human intestinal cancer cell lines. A proliferation inhibition dependent on resveratrol concentration was observed in both cell lines [95].

In another study performed on the intestinal cancer cell lines HCT-116, CO-115 and SW480, after a 24 h incubation with the compound at concentrations ranging from 0 to 50 µM, it was observed that cell viability decreased as the concentration of the compound increased [96].

The effect of resveratrol on the proliferation of neoplastic intestinal cells was also observed in the DLD1 and HCT15 cell lines. Treatment with resveratrol at concentrations ranging from 0 to 40 µM for 24, 48 and 72 h resulted in a dose- and time-dependent cell growth inhibition [97].

In intestinal cancers, resveratrol may act bi-directionally, depending on the concentration. Higher concentrations of the compound result in a significant inhibition of proliferation in particular cell systems, while lower concentrations can stimulate cell growth.

### 5.2. Proapoptotic Effect

Cyclooxygenase 2 (COX-2) overexpression is associated with physiological and pathological processes in the body, including the formation and progression of neoplasms [98,99]. One study compared the levels of COX-2 expression. In the cancer lines HCA-17, SW480 and HT-29, COX-2 was significantly higher compared with the normal cell line CCD-18Co. After 72 h of treatment with resveratrol, a concentration-dependent decrease in COX-2 expression was observed in the intestinal cancer lines. The inhibition of COX-2 expression by resveratrol is frequently associated with an induction of the apoptotic process [93]. After a 24 h incubation of the HT-29 cells with low concentrations of resveratrol (1 and 25 µM), no increase in the number of apoptotic cells was observed. This effect was only visible when higher concentrations of the compound were used [94]. Similar results were noted in studies on the HCT-116, CO-115 and SW480 cell lines. A 24 h incubation with different concentrations of resveratrol (0–50 µM) induced programmed cell death [96].

In intestinal cancer, resveratrol also has proapoptotic properties. This effect is most pronounced when higher concentrations of the compound are used.

## 6. Discussion

Cancer is a complex disease of various aetiologies, and the use of conventional treatments in most cases is associated with numerous side effects. Hence, the current scientific research focuses largely on the search for new therapeutic strategies, especially ones based on compounds of natural origin. High hopes are placed on the possibility of using these compounds in clinical conditions. The most often studied compounds are the polyphenolic compounds, including resveratrol. Due to the diverse range of biological effects, it is considered a promising anticancer agent.

Based on the available scientific literature, in this study we have documented the antitumour effect of resveratrol, especially its role in inhibiting cell proliferation and inducing the apoptotic process.

In vitro studies conducted over the last few decades show that resveratrol can be antiproliferative (decreased cell viability, inhibition of the cell cycle) and proapoptotic in various types of cancer.

In this work, we focused on the effects of resveratrol in cancers of the gastrointestinal tract (pancreatic, stomach, liver and intestinal cancers).

When analysing the scientific reports presented in our study, the effect of resveratrol is strongly dependent on the type of cancer, the concentration of the compound and the duration of its action (Table 1). In the studies examining the effects of resveratrol on pancreatic cancer cells, changes in cell proliferation are seen to occur with higher concentrations of resveratrol (>100 µM), and the most pronounced changes take place after at least 48 h of incubation with the compound.

The effect of resveratrol in stomach cancer (inhibition of cell proliferation) is also specific to certain cells. Changes in cell survival are observed in a wide range of concentrations (0–400 µM). As happens in pancreatic cancer, resveratrol exerts an inhibitory effect on stomach cancer cells in a time-dependent manner.

In liver cancer, the effect of resveratrol on cell proliferation also shows a relationship between the concentration and the duration of the effect. However, in this neoplasm, changes are observed at lower concentrations of the compound (0–100 µM).

A similar relationship between the concentration and the duration of the action of the compound and cell survival can be observed in intestinal cancer. Studies evaluating the antiproliferative properties of resveratrol showed a notable inhibition of cell proliferation in intestinal cancer at significantly lower concentrations of the compound (0–50 µM).

Many of the in vitro studies presented indicate that the antitumor activity of resveratrol may be largely due to the induction of the apoptotic process. Translating the promising results of these in vitro studies into clinical studies is challenging due to the poor pharmacokinetic parameters of the compound—low bioavailability, poor water solubility and fast metabolism [100]—hence, the importance of increasing the bioavailability of resveratrol. For this purpose, delivery systems are being developed to enable compound absorption and an increase in plasma concentration. The methods to increase the bioavailability of resveratrol include, among others, the encapsulation of liposomes or lipid nanocarriers and the development of micelles and emulsions [100,101].

## 7. Conclusions

In in vitro studies, resveratrol elicits a weaker or stronger response depending on the type of cancer. However, in each case there is a correlation between the concentration and time of the action and the effectiveness of the compound. The promising results of the in vitro studies presented in this paper, as well as in many others, emphasize the need for further experiments, especially in vivo, on the effect of resveratrol to determine the therapeutic doses of the compound and the exact mechanisms of its action in relation to different types of cancer.

## Figures and Tables

**Table 1:** List of different cancer cell lines in each type of cancer.

Type of Cancer	Cell Lines	The Most Effective Concentration of Resveratrol (µM)	Antitumor Activity of Resveratrol	Mechanism of Action of Resveratrol	Article
Pancreatic cancer	PANC-1	>100	antiproliferative proapoptotic	Hedgehog signalling pathway up-regulation of Bax protein inhibition of Bcl-2 expression by downgrading miR-21 expression activation of FOXO transcription factors	[57,58,59,61,62]
	AsPC-1				[57,58,59,62]
	BxPC-3				[58,59]
	Capan-2				[60]
	CFPAC-1				[61]
	MIA Paca-2				[61,62]
	Hs766T				[62]
Stomach cancer	MGC803	0–400	antiproliferative	increase in ROS production PTEN/PI3K/Akt pathway inhibition of Wnt signal pathway inhibition of MALAT1-mediated epithelial-to-mesenchymal transition	[70,71]
	SGC-7901		antiproliferative proapoptotic		[72,73,74,75,76]
	BGC823		antiproliferative		[75]
	GES1				
Liver cancer	HepG2	0–100	antiproliferative proapoptotic	up-regulation of Bax protein down-regulation of Bcl-2 protein	[79,80,81,85]
	Bel-7402				[81]
	SMMC-7721				
	MHCC97-H		antiproliferative		[82]
Intestinal cancer	HT-29	0–50	antiproliferative proapoptotic	inhibition of COX-2 expression down-regulation of high telomerase activity (TLMA)	[92,93,94]
	WiDr		antiproliferative		[92]
	HCA-17		antiproliferative proapoptotic		[93]
	SW480				[93,96]
	HCT-116				[94,95,96]
	Caco-2		antiproliferative		[95]
	CO-115		antiproliferative proapoptotic		[96]
	DLD1		antiproliferative		[97]
	HCT15				

## Data Availability

Data sharing not applicable. No new data were created or analyzed in this study. Data sharing is not applicable to this article.

## Sample Availability

Samples of the compounds are not available from the authors.
